# Unique miRNA Expression Profile in MSI- and EMAST-Unstable Sporadic Colon Cancer

**DOI:** 10.3390/genes15081007

**Published:** 2024-08-01

**Authors:** Sonja Marinović, Kristina Vuković Đerfi, Anita Škrtić, Mirko Poljak, Sanja Kapitanović

**Affiliations:** 1 Laboratory for Personalized Medicine, Division of Molecular Medicine, Ruđer Bošković Institute, 10000 Zagreb, Croatia; kvukovic@irb.hr (K.V.Đ.); kapitan@irb.hr (S.K.); 2 Department of Pathology, Clinical Hospital Merkur, 10000 Zagreb, Croatia; skrtic.anita@gmail.com; 3 Department of Surgery, Clinical Hospital Merkur, 10000 Zagreb, Croatia; poljak.mirko@gmail.com

**Keywords:** sporadic colon cancer, MSS/EMAST-S, MSI-H, EMAST-H, miRNA

## Abstract

MicroRNAs (miRNAs) are critical post-transcriptional gene regulators and their involvement in sporadic colon cancer (CRC) tumorigenesis has been confirmed. In this study we investigated differences in miRNA expression in microsatellite stable (MSS/EMAST-S), microsatellite unstable marked by high elevated microsatellite alterations at selected tetranucleotide repeats (MSS/EMAST-H), and high microsatellite unstable (MSI-H/EMAST-H) tumor subgroups as well as in tumors with different clinicopathologic characteristics. An RT-qPCR analysis of miRNA expression was carried out on 45 colon cancer and adjacent normal tissue samples (15 of each group). Overall, we found three differentially expressed miRNAs between the subgroups. miR-92a-3p and miR-224-5p were significantly downregulated in MSI-H/EMAST-H tumors in comparison to other subgroups. miR-518c-3p was significantly upregulated in MSS/EMAST-H tumors in comparison to stable and highly unstable tumors. Furthermore, we showed that miR-143-3p and miR-145-5p were downregulated in tumors in comparison to normal tissues in all subgroups. In addition, we showed overexpression of miR-125b-5p in well-differentiated tumors and miR-451a in less advanced tumors. This is the first report on differences in miRNA expression profiles between MSS/EMAST-S, MSS/EMAST-H, and MSI-H/EMAST-H colorectal cancers. Our findings indicate that the miRNA expression signatures differ in CRC subgroups based on their instability status.

## 1. Introduction

Sporadic colorectal cancer (CRC) is one of the most diagnosed cancers and one of the leading causes of cancer death worldwide [1]. It can develop through two main genetic instability pathways that have different pathological features and clinical outcomes. The chromosomal instability pathway accounts for the majority of CRC cases and it is marked by mutations in the APC and KRAS followed by the inactivation of P53 [2]. The microsatellite instability (MSI) pathway is a result of the inactivation of the DNA mismatch repair (MMR) genes [3,4]. Defects in DNA MMR lead to the development of hypermutable phenotypes that can be observed as characteristic variations in the length of tandem nucleotide repeats that can present the instability in the form of either microsatellite instability (MSI) [4] or elevated microsatellite alterations at selected tetranucleotide repeats (EMAST) [5]. Gene expression analysis allowed the identification of important features of sporadic CRC with MSI such as the methylation of the MLH1 promoter [6], the absence of MLH1 and PMS2 proteins [4] and frequent BRAF mutations [7]. EMAST is, on the other hand, characterized by the suggested loss of function of MSH3 and P53 mutations [4,5,8]. While tumors with MSI have a better prognosis than stable non-MSI tumors, several studies have suggested that EMAST-positive tumors are associated with increased size, higher cancer recurrence, and poor patient outcome [4,8,9].

In recent years, the development of platforms for the analysis of microRNA (miRNA) expression further enriched the molecular classification of CRC [10,11]. miRNAs are short non-coding RNAs that regulate gene expression post-transcriptionally by inhibiting the translation of target mRNAs [12]. They are involved in a variety of cellular processes such as cell cycle regulation, differentiation, proliferation, and apoptosis [12]. The first indication that miRNAs have a role in human disease came from studies in chronic lymphocytic leukemia [13]. Later, it was shown that miRNAs can target oncogenic and tumor suppressor signaling pathways [14] and can therefore influence cancer pathogenesis through changes in proliferation and growth as well as invasion. Furthermore, numerous studies have demonstrated that miRNA expression is dysregulated in different solid tumors [15,16], including colon cancer [17,18].

Up until now, several studies identified differentially expressed miRNAs that have either tumor-suppressing or tumor-promoting activities in CRC. The lethal-7 (let-7) family [19], miR-143-145 cluster [20], and miR-34a [21] have been described to be tumor suppressive miRNAs that decelerate CRC progression through the downregulation of oncogenes such as RAS, c-Myc, and BRAF. On the other hand, miR-21 [22], miR-92 [23], miR-155 [24], and miR-224 [25] have been reported as the most prominent oncogenic miRNAs that mediate cell growth, invasion, migration, stemness, and angiogenesis and therefore accelerate CRC carcinogenesis [19,26]. Even though several studies analyzed miRNA signatures in different subtypes of colorectal cancer, most studies have focused on specific miRNAs and their association with specific CRC subtypes such as P53-mutated tumors or KRAS-mutated tumors. While only a few have looked broadly across the range of miRNAs to identify associations with specific tumor molecular phenotypes [27,28,29] thus far, none of them have included EMAST as a form of instability in miRNA expression analysis. Since EMAST is present in over thirty percent of CRC patients, and it has recently been proposed to be involved in the metastatic spread of colorectal tumors [30], the dysregulation of the miRNA profile might indicate changes in the underlying signaling pathways that contribute to increased metastasis formation and poor prognosis.

Studies examining both the MSI and EMAST status of tumors as a separate entity are rare; therefore, one of the goals of our study was to define different CRC subgroups based on their MSI and/or EMAST instability status. This study proposes that genetic instability pathways, such as MSI and EMAST, are associated with differential expressions of miRNAs in colon cancer that contribute to distinct clinical outcomes. By analyzing miRNA expression profiles in CRC subgroups with different instability statuses, we can identify miRNAs that could play key roles in tumor progression, metastasis, and patient prognosis, thereby uncovering potential biomarkers and therapeutic targets for colon cancer.

## 2. Materials and Methods

### 2.1. Study Subjects and Tissue Samples

Our study included samples from 190 patients with sporadic colon cancer. As previously carried out, colon cancer samples and corresponding adjacent normal tissues located 15 cm from the tumor edge, used as a control, Refs. [31,32,33] were collected from 2016 to 2020 during routine surgery performed in Merkur Clinical Hospital, Zagreb. All samples included in the study were obtained with the patient’s informed consent and were histologically confirmed. A history of prior malignancy and the presence of concurrent malignancy were considered exclusion criteria. In the case where the patients had received previous treatment (e.g., radiation or chemotherapy), had some relevant conditions for this research (e.g., Crohn’s or colitis), or had a known family mutation (e.g., Lynch syndrome), they were excluded from this research. The study was approved by the Ethics Committee of Clinical Hospital Merkur, Zagreb (on 24 May 2016, UR. BR. 03/1-4723), and was conducted according to the ethical standards of the Helsinki Declaration. Fresh tumor samples were stored at −80 °C until DNA and RNA extraction.

### 2.2. MSI and EMAST Analysis

DNA extraction was performed using proteinase K (Invitrogen, Thermo Fischer Scientific, Waltham, MA, USA) digestion and phenol–chloroform (Thermo Scientific Chemicals, Thermo Fischer Scientific, Waltham, MA, USA) extraction from the tumor and corresponding adjacent normal tissues. For both MSI and EMAST analysis, paired normal DNA and tumor DNA were analyzed for changes in five loci each by using a previously published Bethesda panel [34] and a panel of five tetranucleotide markers: D20S82, D20S85, D8S321, D9S242, and MYCL1, respectively. The polymerase chain reaction was performed with previously described primers [35]. A polymorphic analysis of all EMAST and MSI markers was performed by non-denaturing polyacrylamide electrophoresis. Unstable tumor samples had additional bands that differed in length and migrated slightly differently down the gel when compared to corresponding adjacent normal tissues. Samples were considered MSI-stable (MSS) and EMAST-stable (EMAST-S) if instability was not present. They were considered MSI- or EMAST-high (MSI-H and EMAST-H) if two or more markers showed instability and EMAST-low (EMAST-L) if instability was present only in one marker. In our samples, we did not detect the MSI-low (MSI-L) type of instability.

### 2.3. miRNA Extraction and miRNA RT-qPCR

MicroRNA was extracted from snap-frozen samples of resected colon carcinoma and corresponding normal tissues using the mirVana™ miRNA Isolation Kit (Invitrogen, Thermo Fisher Scientific, Waltham, MA, USA, AM1560) according to the manufacturer’s protocol. Isolated RNA tissue samples were normalized to 10 ng of input for reverse transcription and cDNA synthesis was conducted by using the Taqman Advanced miRNA cDNA Synthesis Kit (Applied Biosystems, Thermo Fisher Scientific, Waltham, MA, USA, A28007). For the preliminary analysis of each tumor group (MSS/EMAST-S, MSS/EMAST-L, MSS/EMAST-H, and MSI-H/EMAST-H), we pulled together all 15 samples of prediluted cDNA samples and mixed them with the Taqman Fast Advanced Master Mix (Applied Biosystems, Thermo Fisher Scientific, Waltham, MA, USA, 4444964) that were then added to TaqMan™ Advanced miRNA Human A 96-well plates (Applied Biosystems, Thermo Fisher Scientific, Waltham, MA, USA, A31811). RT-qPCR was performed on the Applied Biosystems QuantStudio 3 (Thermo Fisher Scientific, Waltham, MA, USA) thermocycler with 1 cycle of 95 °C for 20 s, followed by 40 cycles of 95 °C for 1 s denaturation and 60 °C for 20 s. The final copy number per 10 ng RNA was quantified and normalized to determine fold change expression over miR-16.

To validate miRNA expression levels in colon cancer tissues and compare them with corresponding normal tissue expression, we used the Taqman Fast Advanced Master Mix and pre-developed TaqMan™ Advanced miRNA Assays for Human miR-92a-3p (477827_mir), miR-125b-5p (477885_mir), miR-143-3p (477912_mir), miR-145-5p (477916_mir), miR-224-5p (483106_mir), miR-451a (478107_mir), miR-518a-5p (479249_mir), miR-518c-3p (478982_mir), let-7i-5p (478375_mir), and miR-16 (477860_mir) (Applied Biosystems, Thermo Fisher Scientific, Waltham, MA, USA, A25576) as internal controls. The thermal cycling conditions were the same as previously mentioned.

### 2.4. Statistical Analysis

Statistical analysis was performed using the GraphPad Prism 8.4.2. statistical package (GraphPad Software, Boston, MA, USA). To compare the differences between miRNA expressions in corresponding adjacent normal and tumor tissues, a Mann-Whitney U test was used. Kruskal-Wallis test with post hoc Dunn’s multiple comparisons was used to compare miRNA expressions between the groups. Values of * *p* < 0.05, ** *p* < 0.01 and *** *p* < 0.001 and **** *p* < 0.0001 were considered statistically significant.

## 3. Results

### 3.1. Microsatellite Instability Status and Clinicopathological Features of CRC Patients

One hundred and ninety samples of sporadic CRC and corresponding normal colons were examined for the presence of the MSI and EMAST microsatellite instability. Instability was present in 67 (35.3%) tumor samples, while the remaining 123 samples were stable in both MSI and EMAST types of microsatellite instability (MSS/EMAST-S). From unstable tumors, 37 (19.5%) were MSI-stable and showed a low form of EMAST instability (MSS/EMAST-L), 15 (7.9%) were MSI-stable but highly EMAST-unstable (MSS/EMAST-H) and 15 (7.9%) tumors showed high instability in both MSI and EMAST markers (MSI-H/EMAST-H). Clinicopathological features of the patients at the time of surgery are presented in Table 1.

By using χ^2^ and Fisher’s exact test, clinical parameters were compared between stable (MSS/EMAST-S) tumors and tumors that had microsatellite instability (MSS/EMAST-L, MSS/EMAST-H and MSI-H/EMAST-H). The analysis showed a statistically significant difference between MSS/EMAST-S and MSI-H/EMAST-H CRC samples in histological grade and tumor size where poorly differentiated tumors (*p* = 0.015; Table 1) and tumors bigger than 5 cm across (*p* = 0.024; Table 1) were more present in MSI-H/EMAST-H group. In addition, MSI-H/EMAST-H CRCs were more often present in the right colon (*p* < 0.001; Table 1).

### 3.2. miRNA Profile in MSI-H/EMAST-H, MSS/EMAST-H, MSS/EMAST-L, and MSS/EMAST-S CRC

**Figure 1:**
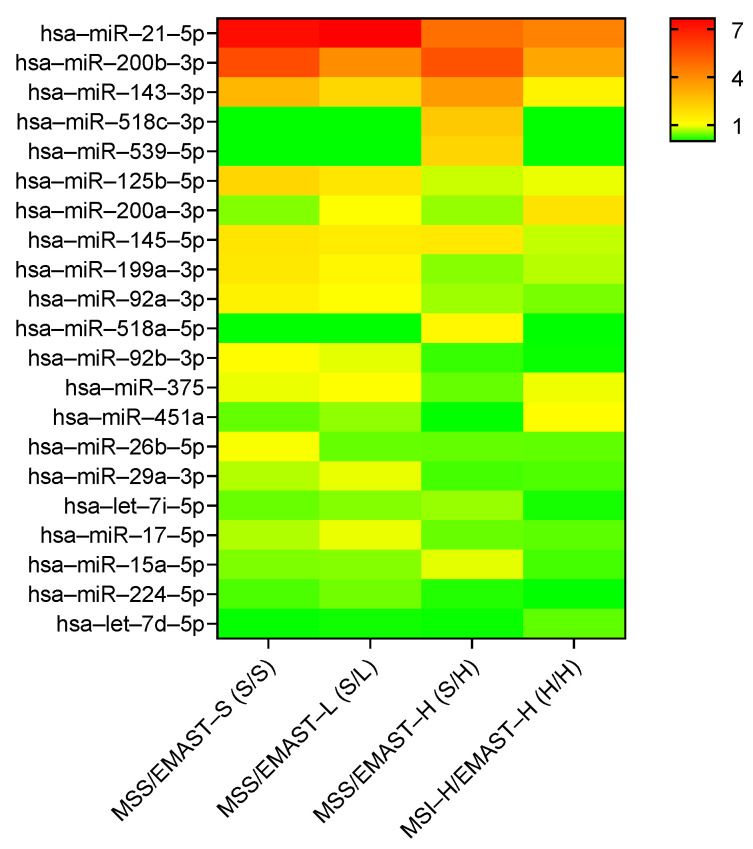
A hierarchical clustering analysis of miRNA expression data obtained from MSS/EMAST-S (S/S), MSS/EMAST-L (S/L), MSS/EMAST-H (S/H) and MSI-H/EMAST-H (H/H) colon cancer tissue samples. A total of 21 miRNAs were identified as differentially expressed between the stable (MSS/EMAST-S) and unstable (MSS/EMAST-L, MSS/EMAST-H, and MSI-H/EMAST-H) tumor tissues. Differentially expressed miRNAs are shown on the left. The CRC groups are shown at the bottom. Red indicates a higher expression of miRNA, and green indicates a lower expression of miRNA.

To investigate the possible difference in miRNA expression profiles between colon cancer subgroups, fifteen tumor samples of each MSS/EMAST-S, MSS/EMAST-L, MSS/EMAST-H, and MSI-H/EMAST-H group were taken to carry out initial miRNA screening on the TaqMan™ Advanced miRNA Human A 96-well plates consisting of 376 miRNA probes. Hierarchical clustering analysis demonstrated a stratification between the miRNA expression profiles of MSI-H/EMAST-H, MSS/EMAST-H, MSS/EMAST-L, and MSS/EMAST-S tumors (Figure 1).

In MSS and EMAST stable (MSS/EMAST-S) and low-EMAST groups, similar miRNA expression patterns were noticed where miR-21-5p, miR-92a-3p, miR-125b-5p, miR-199a-3p, miR-92b-3p, miR-17-5p, and miR-29a-3p were upregulated in comparison to MSS/EMAST-H and MSI-H/EMAST-H. In MSI-H/EMAST-H group, three miRNAs (miR-200a-3p, miR-375-3p, and let-7d) were upregulated and five (miR-143-3p, miR-145-5p, miR-200b-3p, miR-224a-5p, and let-7i-5p) were downregulated when compared to other groups. Lastly, four miRNAs (miR-15a-5p, miR-539-5p, miR-518a-5p, and miR-518c-3p) were upregulated and two (miR-451a and miR-375-3p) were downregulated in the MSS/EMAST-H group (Figure 1). Overall, the initial miRNA screening resulted in the identification of a set of miRNAs capable of discriminating different tumor subtypes based on the presence of microsatellite instability. From a total of 376 miRNAs, 9 differentially expressed miRNAs in MSI and/or EMAST-unstable CRC were singled out that had a fold change of 1.3 or higher or 0.65 or lower relative to MSS/EMAST-S samples.

### 3.3. Validation of Different miRNA Expression Signatures in CRC Subgroups

**Figure 2:**
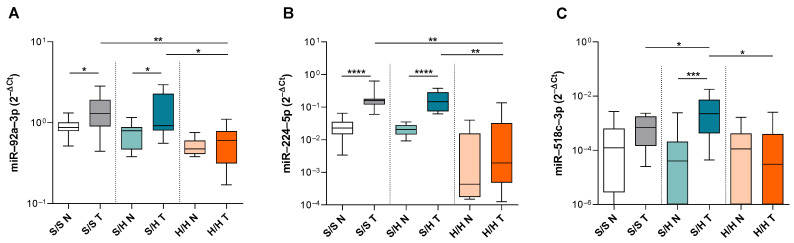
Differences in the expression levels of miR-92a-3p, miR-224a-5p, and miR-518c-3p in the MSS/EMAST-S, MSS/EMAST-H, and MSI-H/EMAST-H subgroups. Tumor tissue (T) and corresponding adjacent normal tissue (N) miRNA expression levels of (**A**) miR-92a-3p, (**B**) miR-224a-5p, and (**C**) miR-518c-3p in the MSS/EMAST-S (S/S), MSS/EMAST-H (S/H) and MSI-H/EMAST-H (H/H) subgroups. Box-and-whisker plots showing the median (horizontal line), 25th and 75th percentiles (box), and highest and lowest values (whiskers). * *p* < 0.05, ** *p* < 0.01, *** *p* < 0.001, **** *p* < 0.0001. The Mann–Whitney U test or Kruskal–Wallis test and the post hoc Dunn’s multiple comparisons test.

To confirm the data obtained from the TaqMan™ Advanced miRNA plates, we quantified the expression of selected miRNAs using real-time RT-PCR assays. Since the MSS/EMAST-S and MSS/EMAST-L groups showed highly similar profiles, we continued our analysis with the MSS/EMAST-S, MSS/EMAST-H, and MSI-H/EMAST-H groups. We measured the expressions of miR-92a-3p, miR-125b-5p, miR-143-3p, miR-145-5p, miR-224a-5p, miR-451a, miR-518a-5p, miR-518c-3p, and let-7i-5p in 15 samples of each tumor subgroup and compared them with their adjacent normal colonic tissue. miRNA expression detected by TaqMan™ Advanced miRNA assays showed that miR-92a-3p and miR-224a-5p had increased expression in the tumor tissue of the MSS/EMAST-S and MSS/EMAST-H subgroups in comparison to normal tissue. Furthermore, both miR-92a-3p and miR-224a-5p were significantly downregulated in MSI-H/EMAST-H tumor tissue in comparison to the tumor tissue of other two subgroups (S/S T vs. H/H T *p* = 0.021; S/H T vs. H/H T *p* = 0.0326 in Figure 2A and S/S T vs. H/H T *p* = 0.001; S/H T vs. H/H T *p* = 0.001 in Figure 2B). In addition, we also found the difference between tumor and normal adjacent tissues in both MSS/EMAST-S and MSS/EMAST-H groups in the expression of miR-92a-3p and miR-224a-5p (S/S N vs. T *p* = 0.023; S/H N vs. T *p* = 0.049 in Figure 2A and S/S N vs. T *p* < 0.0001; S/H N vs. T *p* < 0.0001 in Figure 2B). Notably, miR-518c-3p was significantly upregulated in the MSS/EMAST-H tumor tissue in comparison to corresponding normal tissue and tumor tissues of the MSS/EMAST-S and MSI-H/EMAST-H subgroups (S/H N vs. T *p* = 0.0005; S/S T vs. S/H T *p* = 0.048; S/H T vs. H/H T *p* = 0.003 in Figure 2C).

**Figure 3:**
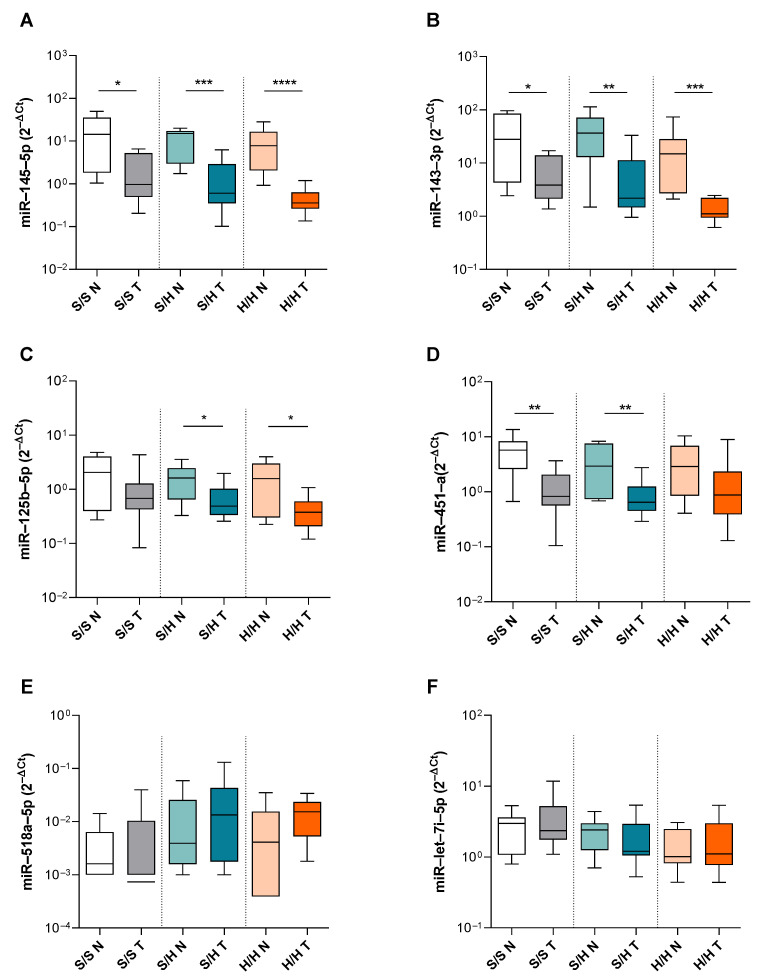
miRNAs expression levels of tumor and corresponding normal tissue in MSS/EMAST-S, MSS/EMAST-H and MSI-H/EMAST-H subgroups. Tumor tissue (T) and corresponding adjacent normal tissue (N) expression levels of (**A**) miR-145-5p, (**B**) miR-143-3p, (**C**) miR-125b-5p, (**D**) miR-451a, (**E**) miR-518a-5p, and (**F**) let-7i-5p in MSS/EMAST-S (S/S), MSS/EMAST-H (S/H) and MSI-H/EMAST-H (H/H) subgroups. Box-and-whisker plots showing the median (horizontal line), 25th and 75th percentiles (box), and highest and lowest values (whiskers). * *p* < 0.05, ** *p* < 0.01, *** *p* < 0.001, **** *p* < 0.0001. Mann–Whitney U test.

The miR-143-3p and miR-145-5p clusters, which have been widely described as tumor suppressors, had decreased expression in tumor tissue in comparison to normal tissue in all three subgroups (S/S N vs. T *p* = 0.023; S/H N vs. T *p* = 0.001 and H/H N vs. T *p* < 0.0001 in Figure 3A, S/S N vs. T *p* = 0.049; S/H N vs. T *p* = 0.004 and H/H N vs. T *p* = 0.0002 in Figure 3B). In addition, miR-125b-5p was downregulated in the unstable tumor tissues of the MSS/EMAST-H and MSI-H/EMAST-H subgroups (S/H N vs. T *p* = 0.043 and H/H N vs. T *p* = 0.036 in Figure 3C) and miR-451a was downregulated in the tumor tissues of the MSS/EMAST-S and MSS/EMAST-H subgroups (S/S N vs. T *p* = 0.001; S/H N vs. T *p* = 0.009 in Figure 3D), while miR-518a-5p and let-7i-5p showed no differences between the subgroups (Figure 3E,F). In total, seven miRNAs were dysregulated in tumor tissues in comparison to normal tissues, with two miRNAs being dysregulated in all subgroups. Overall, we were able to partially validate the obtained TaqMan™ Advanced miRNA plate results and we showed different expressions of three miRNAs (miR-518c-3p, miR-92a-3p, and miR-224a-5p) between the tumor subgroups.

### 3.4. Association of Relative miRNA Expression with Clinicopathological Characteristics

**Figure 4:**
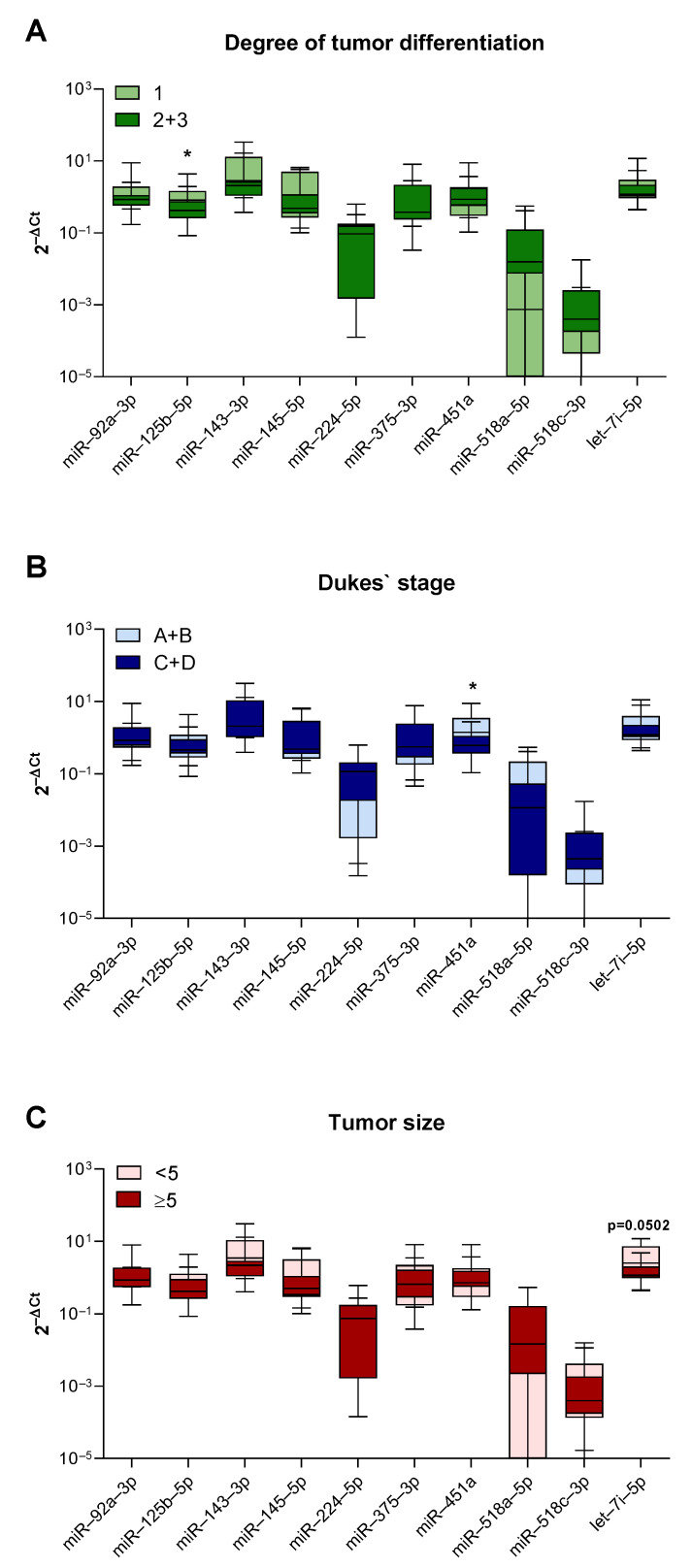
Correlation between miRNA expression levels and clinicopathological features. Expression levels of miR-92a-3p, miR-125b-5p, miR-143-3p, miR-145-5p, miR-224a-5p, miR-451a, miR-518a-5p, miR-518c-3p and let-7i-5p in tumor tissues and correlation with (**A**) tumor differentiation, (**B**) Dukes’ stage, and (**C**) tumor size. Box-and-whisker plots showing the median (horizontal line), 25th and 75th percentiles (box), and highest and lowest values (whiskers). * *p* < 0.05. Mann–Whitney U test.

In addition to differences between the CRC subgroups, we carried out statistical analysis between nine selected miRNAs and several clinicopathological characteristics. Additional observations from these comparisons were a statistically significant increase in the expression of miR-125b-5p in well-differentiated (grade 1) tumors (*p* = 0.045, Figure 4A) and an increased expression of miR-451a in less advanced (Dukes’ A+B) tumors (*p* = 0.027, Figure 4B). We also observed an increased expression of let-7i-5p in smaller tumors (less than 5 cm across) (*p* = 0.0502, Figure 4C); however, this result did not reach statistical significance.

## 4. Discussion

Since colorectal tumors characterized by MSI and EMAST types of instability are distinct from MSS/EMAST-S tumors in many molecular aspects, we considered that their miRNA expression patterns could be affected by MLH1 methylation, a higher frequency of BRAF mutations, MSH3 dysfunction, and lower frequencies of APC, KRAS, and TP53 mutations. In our two-step investigation of miRNA expression, we first found 21 differentially expressed miRNAs between groups. In the second step, we evaluated nine selected miRNAs and identified three miRNAs (miR-92a-3p, miR-224-5p, and miR-518c-3p) that have different expression patterns in MSS/EMAST-S, MSS/EMAST-H, and MSI-H/EMAST-H tumors.

In the initial analysis, using TaqMan™ Advanced miRNA plates with 376 targets, we found several miRNAs that had different expressions in the tumor tissues of unstable MSS/EMAST-H and MSI-H/EMAST-H subgroups in comparison to stable MSS/EMAST-S tumors. Therefore, we tried to confirm this finding by subsequent miRNA assay analysis. We found two miRNAs, miR-92a-3p and miR-224-5p, that were significantly downregulated in the tumor tissue of the MSI-H/EMAST-H subgroup in comparison to stable MSS/EMAST-S and unstable MSS/EMAST-H tumors. The increased expression of miR-92a-3p has already been observed in the tumor tissues of patients with CRC [23], and it has been shown that the miR-92 family regulates the formation of vascular endothelial cells and blood vessels and thus can have an important oncogenic role in CRC [36]. Furthermore, it was shown that miR-92a-3p can activate the Wnt/β-catenin signaling pathway and consequently promote the stem-like phenotype of colorectal cancer cells [37]. Conversely, miR-92a-3p inhibition results in reduced proliferation and invasion, as well as induced cell death [38,39]. Interestingly, our study showed miR-92a-3p to be overexpressed only in the tumor tissues of MSS/EMAST-S and MSS/EMAST-H but not MSI-H/EMAST-H CRC patients. This result is in line with previously published studies that have identified decreased miR-92a expression in unstable tumors [28,29]. Hence, it is possible that decreased miR-92a-3p expression could limit Wnt/β-catenin signaling and decrease the possibility of metastasis formation, both effects often seen in MSI-H/EMAST-H tumors that have been proven to have a better prognosis [40,41,42,43]. Besides miR-92a-3p, miR-224 was also previously reported to be overexpressed in CRC and associated with an aggressive phenotype and poor prognosis through the regulation of SMAD4 and p21 [25,44]. Moreover, just recently, it was published that miR-224 levels increase gradually along the CRC invasion–metastasis cascade [45]. In addition, the same group showed that miR-224 expression is lower in stage IV BRAF-mutated and dMMR tumors [45], an effect we also observed in MSI-H/EMAST-H tumors, irrespective of the tumor stage. This could suggest that miR-224 is involved in the acquisition of the metastatic phenotype in MSS/EMAST-S tumors and it would be of interest to further study this. In our initial study, we also confirmed results for several miRNAs investigated for association with the MSI status of CRC such as miR-17-5p and miR-26b. Nevertheless, several miRNAs (miR-223, miR-155, miR-196a, and miR-31 [29]) that have previously been suggested to correlate with MSI-H status were not deregulated in our MSI-H/EMAST-H subgroup. There are several possible explanations for these differences. First, we included in our analysis a third group of tumors, the MSS/EMAST-H subtype. In addition, different tissue procurement and methods were used. Finally, there could be differences in the relative amount of tumor and “contaminating” adjacent tissue such as inflammatory cells, which are often plentiful in MSI-H CRC [46].

Furthermore, we found three (miR-518c-3p, miR-518a-5p, and miR-539-5p) differentially expressed miRNA in MSS/EMAST-H tumors in our initial study. miR-518c-3p has been shown to inhibit cell proliferation, invasiveness, and migration in CRC cell lines, all of which suggest a tumor-suppressor function [47]. Yet, Tay et al. showed in vitro that miR-518c-3p can target and inactivate both TP53 and PTEN genes in multiple human cancer cell lines which would advocate the opposing oncogenic role [48]. Our results showed an increased expression in MSS/EMAST-H tumors in comparison to MSS/EMAST-S and MSI-H/EMAST-H tumors and adjacent normal tissue. Since EMAST-positive tumors have been associated with poor prognosis [49], these results would further support the oncogenic role of miR-518c-3p in sporadic CRC. Even though differences in miR-518a-5p expression did not reach statistical significance, we could see a trend in the higher expression of this miRNA in MSS/EMAST-H tumors in comparison to other groups. Interestingly, both of these miRNAs are involved in the regulation of the TGF-β signaling pathway that plays a role in tumor progression and metastasis formation [50]. Since patients with EMAST cancers present with an advanced stage and are more likely to have metastasis [48], it is possible that these miRNAs might play a role in the regulation of the TGF-β signaling pathway in MSS/EMAST-H tumors.

In line with previous reports, our results also showed that several miRNAs are aberrantly expressed in colon cancer relative to normal tissue. For instance, in all three subgroups, tumor suppressor miRNAs, miR-143-3p and miR-145-5p, were downregulated and showed statistically significant differences between tumor and normal tissue as previously reported [51,52]. Even though miR-125-5p was previously reported to be downregulated in CRC [53], we observed a statistically significant difference only in unstable tumors, even though the trend was also observed in stable tumors. Interestingly, miR-451a was downregulated in MSS/EMAST-S and MSS/EMAST-H but not MSI-H/EMAST-H tumors, which might have a certain prognostic significance since it was shown that miR-451a can inhibit colon cancer growth through the downregulation of the P13K/AKT pathway [54].

Since findings from multiple studies [55], but also our results, showed that dMMR status is associated with poor tumor differentiation, tumor location and size, the expression levels of all nine miRNAs were correlated with the tumor size, histological grade, and Dukes’ stage. The results showed a trend, but did not reach statistical significance, in the increased expression of let-7i-5p in smaller tumors, which goes in line with the consideration that miRNAs from the let-7 family are tumor suppressors [56] and have been shown to be significantly downregulated in ovarian and bladder advanced tumors [57,58]. However, we showed that miR-125b-5p had increased expression in well-differentiated tumors. Even though molecular mechanisms defining the histological grade remain unclear, poor histological differentiation is still considered an unfavorable hallmark of CRC. And while miR-125b has a controversial role in different malignant diseases [59], our results might relate to Cenariu et al., who showed in vitro that miR-125b can diminish CRC malignant behavior [35]. Lastly, we noticed an increased expression of miR-451a in less advanced tumors classified as Dukes’ A and B, which would be in accordance with the previously mentioned potential tumor suppressor role of miR-451a [54].

In conclusion, the findings of our study reinforce those of previous publications on miRNA expression in CRC but also identify differences in miRNA expression in relation to MSI-H and EMAST-H statuses that have not been previously reported. To the best of our knowledge, we are the first to describe the miRNA profile of the EMAST-H subgroup of CRC. Even though these findings and their potential roles as molecular classifiers and/or clinical biomarkers will require further validation in larger cohort studies, we hope that our results will improve the current knowledge in the molecular profiling of sporadic CRC.

## Figures and Tables

**Table 1:** Clinicopathological features of 190 CRC patients included in the study.

Characteristics	MSS/EMAST-S ^a^ *n* = 123(64.7%)	MSS/EMAST-L ^b^ *n* = 37 (19.5%)	MSS/EMAST-H ^c^ *n* = 15 (7.9%)	MSI-H/EMAST-H ^d^ *n* = 15 (7.9%)	*p*
Age					^a^ vs. ^b^ 0.442
≤60	44 (38.5)	16 (43.2)	3 (20.0)	3 (20.0)	^a^ vs. ^c^ 0.264
>60	79 (64.2)	21 (56.8)	12 (80.0)	12 (80.0)	^a^ vs. ^d^ 0.264
Sex					^a^ vs. ^b^ 0.326
Male	77 (62.6)	27 (73.0)	9 (60.0)	6 (40.0)	^a^ vs. ^c^ 0.999
Female	46 (37.4)	10 (27.0)	6 (40.0)	9 (60.0)	^a^ vs. ^d^ 0.102
Tumor size					^a^ vs. ^b^ 0.850
≤5 cm	74 (60.1)	23 (62.2)	7 (46.7)	4 (26.7)	^a^ vs. ^c^ 0.406
>5 cm	49 (39.9)	14 (37.8)	8 (53.3)	11 (73.3)	^a^ vs. ^d^ 0.024 *
Tumor location					^a^ vs. ^b^ 0.054
Left colon	100 (81.3)	24 (64.9)	9 (60.0)	2 (13.3)	^a^ vs. ^c^ 0.087
Right colon	23 (18.7)	13 (35.1)	6 (40.0)	13 (86.7)	^a^ vs. ^d^ <0.001 ***
Dukes’ stage					
A	10 (8.1)	1 (2.7)	0 (0)	0 (0)	
B	33 (26.9)	13 (35.1)	4 (26.7)	8 (53.3)	^a^ vs. ^b^ 0.089
C	50 (40.7)	13 (35.1)	7 (46.6)	5 (33.4)	^a^ vs. ^c^ 0.714
D	30 (24.3)	10 (27.1)	4 (26.7)	2 (13.3)	^a^ vs. ^d^ 0.153
Histological grade					
Well	49 (39.8)	14 (37.8)	6 (40.0)	3 (20.0)	^a^ vs. ^b^ 0.448
Moderate	63 (51.3)	17 (45.9)	6 (40.0)	7 (46.7)	^a^ vs. ^c^ 0.377
Poor	11 (8.9)	6 (16.3)	3 (20.0)	5 (33.3)	^a^ vs. ^d^ 0.015 *

^a^ vs. ^b^ indicates the difference between MSS/EMAST-S and MSS/EMAST-L, ^a^ vs. ^c^ indicates the difference between MSS/EMAST-S and MSS/EMAST-H, ^a^ vs. ^d^ indicates the difference between MSS/EMAST-S and MSI-H/EMAST-H. *p*-values were obtained by Fisher’s exact test. * *p* < 0.05, *** *p* < 0.001.

## Data Availability

The original contributions presented in the study are included in the article, further inquiries can be directed to the corresponding authors.

## References

[1] Xi Y., Xu P. (2021). Global colorectal cancer burden in 2020 and projections to 2040. Transl. Oncol..

[2] Pino M.S., Chung D.C. (2010). The chromosomal instability pathway in colon cancer. Gastroenterology.

[3] Kunkel T.A., Erie D.A. (2015). Eukaryotic Mismatch Repair in Relation to DNA Replication. Annu. Rev. Genet..

[4] Boland C.R., Goel A. (2010). Microsatellite instability in colorectal cancer. Gastroenterology.

[5] Watson M.M., Lea D., Hagland H.R., Soreide K. (2019). Elevated Microsatellite Alterations at Selected Tetranucleotides (EMAST) Is Not Attributed to MSH3 Loss in Stage I-III Colon cancer: An Automated, Digitalized Assessment by Immunohistochemistry of Whole Slides and Hot Spots. Transl. Oncol..

[6] Veigl M.L., Kasturi L., Olechnowicz J., Ma A.H., Lutterbaugh J.D., Periyasamy S., Li G.M., Drummond J., Modrich P.L., Sedwick W.D. (1998). Biallelic inactivation of hMLH1 by epigenetic gene silencing, a novel mechanism causing human MSI cancers. Proc. Natl. Acad. Sci. USA.

[7] Wang L., Cunningham J.M., Winters J.L., Guenther J.C., French A.J., Boardman L.A., Burgart L.J., McDonnell S.K., Schaid D.J., Thibodeau S.N. (2003). BRAF mutations in colon cancer are not likely attributable to defective DNA mismatch repair. Cancer Res..

[8] Haugen A.C., Goel A., Yamada K., Marra G., Nguyen T.P., Nagasaka T., Kanazawa S., Koike J., Kikuchi Y., Zhong X. (2008). Genetic instability caused by loss of MutS homologue 3 in human colorectal cancer. Cancer Res..

[9] Vukovic Derfi K., Salar A., Cacev T., Kapitanovic S. (2023). EMAST Type of Microsatellite Instability-A Distinct Entity or Blurred Overlap between Stable and MSI Tumors. Genes.

[10] Zhu J., Xu Y., Liu S., Qiao L., Sun J., Zhao Q. (2020). MicroRNAs Associated with Colon Cancer: New Potential Prognostic Markers and Targets for Therapy. Front. Bioeng. Biotechnol..

[11] Wang J.Y., Wang C.L., Wang X.M., Liu F.J. (2017). Comprehensive analysis of microRNA/mRNA signature in colon adenocarcinoma. Eur. Rev. Med. Pharmacol. Sci..

[12] Croce C.M., Calin G.A. (2005). miRNAs, cancer, and stem cell division. Cell.

[13] Calin G.A., Dumitru C.D., Shimizu M., Bichi R., Zupo S., Noch E., Aldler H., Rattan S., Keating M., Rai K. (2002). Frequent deletions and down-regulation of micro- RNA genes miR15 and miR16 at 13q14 in chronic lymphocytic leukemia. Proc. Natl. Acad. Sci. USA.

[14] He L., He X., Lowe S.W., Hannon G.J. (2007). microRNAs join the p53 network—Another piece in the tumour-suppression puzzle. Nat. Rev. Cancer.

[15] Fridrichova I., Zmetakova I. (2019). MicroRNAs Contribute to Breast Cancer Invasiveness. Cells.

[16] Iqbal M.A., Arora S., Prakasam G., Calin G.A., Syed M.A. (2019). MicroRNA in lung cancer: Role, mechanisms, pathways and therapeutic relevance. Mol. Asp. Med..

[17] Sahu S.S., Dey S., Nabinger S.C., Jiang G., Bates A., Tanaka H., Liu Y., Kota J. (2019). The Role and Therapeutic Potential of miRNAs in Colorectal Liver Metastasis. Sci. Rep..

[18] Schetter A.J., Okayama H., Harris C.C. (2012). The role of microRNAs in colorectal cancer. Cancer J..

[19] Ding L., Lan Z., Xiong X., Ao H., Feng Y., Gu H., Yu M., Cui Q. (2018). The Dual Role of MicroRNAs in Colorectal Cancer Progression. Int. J. Mol. Sci..

[20] Meng W.J., Yang L., Ma Q., Zhang H., Adell G., Arbman G., Wang Z.Q., Li Y., Zhou Z.G., Sun X.F. (2015). MicroRNA Expression Profile Reveals miR-17-92 and miR-143-145 Cluster in Synchronous Colorectal Cancer. Medicine.

[21] Li Y., Zeng C., Hu J., Pan Y., Shan Y., Liu B., Jia L. (2018). Long non-coding RNA-SNHG7 acts as a target of miR-34a to increase GALNT7 level and regulate PI3K/Akt/mTOR pathway in colorectal cancer progression. J. Hematol. Oncol..

[22] Valeri N., Gasparini P., Braconi C., Paone A., Lovat F., Fabbri M., Sumani K.M., Alder H., Amadori D., Patel T. (2010). MicroRNA-21 induces resistance to 5-fluorouracil by down-regulating human DNA MutS homolog 2 (hMSH2). Proc. Natl. Acad. Sci. USA.

[23] Zhang G., Zhou H., Xiao H., Liu Z., Tian H., Zhou T. (2014). MicroRNA-92a functions as an oncogene in colorectal cancer by targeting PTEN. Dig. Dis. Sci..

[24] Gironella M., Seux M., Xie M.J., Cano C., Tomasini R., Gommeaux J., Garcia S., Nowak J., Yeung M.L., Jeang K.T. (2007). Tumor protein 53-induced nuclear protein 1 expression is repressed by miR-155, and its restoration inhibits pancreatic tumor development. Proc. Natl. Acad. Sci. USA.

[25] Liao W.T., Li T.T., Wang Z.G., Wang S.Y., He M.R., Ye Y.P., Qi L., Cui Y.M., Wu P., Jiao H.L. (2013). microRNA-224 promotes cell proliferation and tumor growth in human colorectal cancer by repressing PHLPP1 and PHLPP2. Clin. Cancer Res..

[26] Catela Ivkovic T., Voss G., Cornella H., Ceder Y. (2017). microRNAs as cancer therapeutics: A step closer to clinical application. Cancer Lett..

[27] Zhang N., Hu X., Du Y., Du J. (2021). The role of miRNAs in colorectal cancer progression and chemoradiotherapy. Biomed. Pharmacother..

[28] Lanza G., Ferracin M., Gafa R., Veronese A., Spizzo R., Pichiorri F., Liu C.G., Calin G.A., Croce C.M., Negrini M. (2007). mRNA/microRNA gene expression profile in microsatellite unstable colorectal cancer. Mol. Cancer.

[29] Earle J.S., Luthra R., Romans A., Abraham R., Ensor J., Yao H., Hamilton S.R. (2010). Association of microRNA expression with microsatellite instability status in colorectal adenocarcinoma. J. Mol. Diagn..

[30] Torshizi Esfahani A., Mohammadpour S., Jalali P., Yaghoobi A., Karimpour R., Torkamani S., Pardakhtchi A., Salehi Z., Nazemalhosseini-Mojarad E. (2024). Differential Expression of Angiogenesis-Related Genes ‘Vegf’ and ‘Angiopoietin-1’ in Metastatic and Emast-Positive Colorectal Cancer Patients. Sci. Rep..

[31] Marinovic S., Vukovic K., Skrtic A., Poljak M., Petek S., Petek L., Kapitanovic S. (2021). Epidermal growth factor receptor intron 1 polymorphism and microsatellite instability in sporadic colorectal cancer. Oncol. Lett..

[32] Suto T., Yokobori T., Yajima R., Morita H., Fujii T., Yamaguchi S., Altan B., Tsutsumi S., Asao T., Kuwano H. (2015). MicroRNA-7 expression in colorectal cancer is associated with poor prognosis and regulates cetuximab sensitivity via EGFR regulation. Carcinogenesis.

[33] Sun Y., Shen S., Liu X., Tang H., Wang Z., Yu Z., Li X., Wu M. (2014). MiR-429 inhibits cells growth and invasion and regulates EMT-related marker genes by targeting Onecut2 in colorectal carcinoma. Mol. Cell Biochem..

[34] Murphy K.M., Zhang S., Geiger T., Hafez M.J., Bacher J., Berg K.D., Eshleman J.R. (2006). Comparison of the microsatellite instability analysis system and the Bethesda panel for the determination of microsatellite instability in colorectal cancers. J. Mol. Diagn..

[35] Cenariu D., Zimta A.A., Munteanu R., Onaciu A., Moldovan C.S., Jurj A., Raduly L., Moldovan A., Florea A., Budisan L. (2021). Hsa-miR-125b Therapeutic Role in Colon Cancer Is Dependent on the Mutation Status of the TP53 Gene. Pharmaceutics.

[36] Deng S., Zhang Y., Wang Y., Lu X., Jiang Q. (2019). MicroRNA-92 regulates vascular smooth muscle cell function by targeting KLF4 during vascular restenosis and injury. Int. J. Clin. Exp. Pathol..

[37] Zhang G.J., Li L.F., Yang G.D., Xia S.S., Wang R., Leng Z.W., Liu Z.L., Tian H.P., He Y., Meng C.Y. (2017). MiR-92a promotes stem cell-like properties by activating Wnt/beta-catenin signaling in colorectal cancer. Oncotarget.

[38] Yamada N., Nakagawa Y., Tsujimura N., Kumazaki M., Noguchi S., Mori T., Hirata I., Maruo K., Akao Y. (2013). Role of Intracellular and Extracellular MicroRNA-92a in Colorectal Cancer. Transl. Oncol..

[39] Ahmadi S., Sharifi M., Salehi R. (2016). Locked nucleic acid inhibits miR-92a-3p in human colorectal cancer, induces apoptosis and inhibits cell proliferation. Cancer Gene Ther..

[40] Oh B.Y., Huh J.W., Park Y.A., Cho Y.B., Yun S.H., Kim H.C., Lee W.Y., Chun H.K. (2016). Prognostic factors in sporadic colon cancer with high-level microsatellite instability. Surgery.

[41] Fujiyoshi K., Yamamoto G., Takenoya T., Takahashi A., Arai Y., Yamada M., Kakuta M., Yamaguchi K., Akagi Y., Nishimura Y. (2017). Metastatic Pattern of Stage IV Colorectal Cancer with High-Frequency Microsatellite Instability as a Prognostic Factor. Anticancer. Res..

[42] Wang H., Wang X., Xu L., Zhang J., Cao H. (2019). Analysis of the transcriptomic features of microsatellite instability subtype colon cancer. BMC Cancer..

[43] Cercek A., Lumish M., Sinopoli J., Weiss J., Shia J., Lamendola-Essel M., El Dika I.H., Segal N., Shcherba M., Sugarman R. (2022). PD-1 Blockade in Mismatch Repair-Deficient, Locally Advanced Rectal Cancer. N. Engl. J. Med..

[44] Ling H., Pickard K., Ivan C., Isella C., Ikuo M., Mitter R., Spizzo R., Bullock M., Braicu C., Pileczki V. (2016). The clinical and biological significance of MIR-224 expression in colorectal cancer metastasis. Gut.

[45] Fassan M., Cui R., Gasparini P., Mescoli C., Guzzardo V., Vicentini C., Munari G., Loupakis F., Lonardi S., Braconi C. (2019). miR-224 Is Significantly Upregulated and Targets Caspase-3 and Caspase-7 During Colorectal Carcinogenesis. Transl. Oncol..

[46] Lin A., Zhang J., Luo P. (2020). Crosstalk Between the MSI Status and Tumor Microenvironment in Colorectal Cancer. Front. Immunol..

[47] Yang H., Ren J., Bai Y., Jiang J., Xiao S. (2020). MicroRNA-518-3p suppresses cell proliferation, invasiveness, and migration in colorectal cancer via targeting TRIP4. Biochem. Cell Biol..

[48] Tay Y., Tan S.M., Karreth F.A., Lieberman J., Pandolfi P.P. (2014). Characterization of dual PTEN and p53-targeting microRNAs identifies microRNA-638/Dnm2 as a two-hit oncogenic locus. Cell Rep..

[49] Garcia M., Choi C., Kim H.R., Daoud Y., Toiyama Y., Takahashi M., Goel A., Boland C.R., Koi M. (2012). Association between recurrent metastasis from stage II and III primary colorectal tumors and moderate microsatellite instability. Gastroenterology.

[50] Pellatt A.J., Mullany L.E., Herrick J.S., Sakoda L.C., Wolff R.K., Samowitz W.S., Slattery M.L. (2018). The TGFbeta-signaling pathway and colorectal cancer: Associations between dysregulated genes and miRNAs. J. Transl. Med..

[51] Jin Y.P., Hu Y.P., Wu X.S., Wu Y.S., Ye Y.Y., Li H.F., Liu Y.C., Jiang L., Liu F.T., Zhang Y.J. (2018). miR-143-3p targeting of ITGA6 suppresses tumour growth and angiogenesis by downregulating PLGF expression via the PI3K/AKT pathway in gallbladder carcinoma. Cell Death Dis..

[52] Yang M., Tang X., Wang Z., Wu X., Tang D., Wang D. (2019). miR-125 inhibits colorectal cancer proliferation invasion by targeting, TAZ. Biosci. Rep..

[53] Cheng X., Shen T., Liu P., Fang S., Yang Z., Li Y., Dong J. (2022). mir-145-5p is a suppressor of colorectal cancer at early stage, while promotes colorectal cancer metastasis at late stage through regulating AKT signaling evoked EMT-mediated anoikis. BMC Cancer.

[54] Bai H., Wu S. (2019). miR-451: A Novel Biomarker and Potential Therapeutic Target for Cancer. Onco Targets Ther..

[55] Mei W.J., Mi M., Qian J., Xiao N., Yuan Y., Ding P.R. (2022). Clinicopathological characteristics of high microsatellite instability/mismatch repair-deficient colorectal cancer: A narrative review. Front. Immunol..

[56] Calin G.A., Sevignani C., Dumitru C.D., Hyslop T., Noch E., Yendamuri S., Shimizu M., Rattan S., Bullrich F., Negrini M. (2004). Human microRNA genes are frequently located at fragile sites and genomic regions involved in cancers. Proc. Natl. Acad. Sci. USA.

[57] Yang N., Kaur S., Volinia S., Greshock J., Lassus H., Hasegawa K., Liang S., Leminen A., Deng S., Smith L. (2008). MicroRNA microarray identifies Let-7i as a novel biomarker and therapeutic target in human epithelial ovarian cancer. Cancer Res..

[58] Qin M.M., Chai X., Huang H.B., Feng G., Li X.N., Zhang J., Zheng R., Liu X.C., Pu C. (2019). let-7i inhibits proliferation and migration of bladder cancer cells by targeting HMGA1. BMC Urol..

[59] Banzhaf-Strathmann J., Edbauer D. (2014). Good guy or bad guy: The opposing roles of microRNA 125b in cancer. Cell Commun. Signal.

